# A systematic review on dysphagia treatments for persons living with dementia

**DOI:** 10.1007/s41999-024-01107-6

**Published:** 2024-11-29

**Authors:** Charis Tsz Wun Chan, Tsz Yin Wu, Ivy Cheng

**Affiliations:** 1 Academic Unit of Human Communication, Learning and Development, Faculty of Education, The University of Hong Kong, Room 766, 7/F, Meng Wah Complex, Hong Kong, China; 2 Centre for Gastrointestinal Sciences, Division of Diabetes, Endocrinology and Gastroenterology, School of Medical Sciences, Faculty of Biology, Medicine and Health, The University of Manchester, Manchester, UK

**Keywords:** Dementia, Dysphagia, Geriatric, Systematic review, Treatment

## Abstract

**Purpose:**

Dysphagia in persons living with dementia (PLWD) is raising concern due to the absence of swallowing treatments specific to this population. Dysphagia can result in detrimental effects, such as malnutrition, dehydration, and aspiration pneumonia. This systematic review aimed to evaluate the existing literature on dysphagia management of PLWD and reveal their limitations to facilitate clinical practitioners in decision-making.

**Methods:**

Five electronic databases were searched systematically from the date of inception to January 2024 by two independent reviewers. Eligible studies investigated any treatments with outcome measurements evaluating swallowing function. Qualitative analyses were performed.

**Results:**

A total of 10 studies, involving 1360 participants, were included in the systematic review. They were analyzed qualitatively and evaluated for quality. Studies included were observational (*n* = 5) with prospective or retrospective designs, and randomized controlled trials (*n* = 5). Compensatory, rehabilitative, stimulation, and pharmacological treatments were addressed.

**Conclusion:**

There is currently limited evidence available revealing the efficacy of dysphagia treatments for PLWD. Based on the current evidence, no definitive conclusions can be drawn on which treatments are more effective for this population. In the future, more randomized controlled trials with large sample sizes investigating the long-term effects of treatments are warranted.

**Supplementary Information:**

The online version contains supplementary material available at 10.1007/s41999-024-01107-6.

## Key summary points

**Aim:**

To evaluate the existing literature on dysphagia management of persons living with dementia (PLWD) to facilitate clinical practitioners in decision-making.

**Findings:**

There is currently limited evidence available revealing the efficacy of dysphagia treatments for PLWD. No definitive conclusions can be drawn on which treatments are more effective for this population based on the current evidence.

**Message:**

There is an urgent need for systematic investigations through randomized controlled trials on the clinical efficacy of dysphagia treatments for PLWD.

**Supplementary Information:**

The online version contains supplementary material available at 10.1007/s41999-024-01107-6.

## Introduction

Dementia is a progressive neurological disorder caused by neurological disease or injury and is characterized by a decline in cognitive abilities (e.g., memory, thinking, judgment, language, processing, orientation, attention, planning), which affects daily life functioning to different extents depending on the severity [1, 2]. Globally, at least 55 million people are affected by dementia, and it is the seventh major cause of mortality and disability in older adults [2]. Alzheimer’s disease (AD), vascular dementia, Lewy body dementia, and frontotemporal dementia are common types of dementia, with AD contributing to 60–70% of dementia cases [2, 3].

Persons living with dementia (PLWD) may experience dysphagia during the moderate and late stages of the disease [4–6]. The prevalence of oropharyngeal dysphagia among PLWD is up to 93% upon instrumental assessment [7]. Dysphagia refers to difficulties in transferring food from the oral cavity to the stomach safely and effectively [8]. During the late stages of dementia, PLWD may lose the ability to recognize food and their eating behavior can be adversely altered [9]. PLWD may also experience reduced food intake and appetite [10], prolonged oral and pharyngeal phase of swallowing, atypical and reduced mastication, tongue and jaw movements, and forgetting to swallow [11].

Dysphagia can lead to weight loss, dehydration, malnutrition, aspiration pneumonia, chronic lung disease, and death [12]. A recent study has shown that dysphagia is associated with dehydration in PLWD [13]. Traditional dysphagia treatments are divided into compensatory and rehabilitative approaches. Compensatory techniques, which includes modifying food consistencies or feeding posture, aim to improve swallowing activity without causing long-term changes to the physiology. Rehabilitative approaches such as swallowing and muscle strengthening exercises or sensory stimulation aim for sustainable improvement in swallowing function [8].

Currently, there is no dysphagia treatment specific for PLWD. Although much research has focused on dysphagia treatments for stroke and Parkinson’s disease [14, 15], available studies on dysphagia treatments for PLWD are scarce, most of which are not of the highest level of evidence (i.e., randomized controlled trials [RCT]), indicating limited treatment options for PLWD. Ashford et al. [16] conducted a systematic review on dysphagia treatments for neurological disorders, but it only included behavioral treatments and excluded studies with combined treatments. Moreover, this review lacked specificity as it also included participants with brain injury, stroke, and Parkinson’s disease. The systematic review by Alagiakrishnan et al. [12] addressed the issue of excluding combined treatments by focusing on both swallowing assessments and treatments for different types of dementia but concluded that much evidence is needed to make further conclusions regarding treatment effectiveness. However, the review was conducted over a decade ago, suggesting the need for an updated review.

With the rapidly aging population, the prevalence of dementia and PLWD’s demand for healthcare services are anticipated to rise [3]. The need for a systematic organization of the current evidence on swallowing treatments for PLWD is pressing. Therefore, this review aims to provide an up-to-date evaluation on the clinical efficacy of dysphagia treatments (including combined treatments) based on existing literature. The findings will aid clinical practitioners in decision-making and improving cost-effectiveness in healthcare management.

## Methods

The reporting of this systematic review followed the Preferred Reporting Items for Systematic Reviews and Meta-Analyses (PRISMA) guidelines [17], and the protocol was registered and approved by PROSPERO, an international database of systematic review protocols (registration ID: CRD42023485030).

### Search method

The literature search was done independently by the first and second authors. Literature was searched from five electronic databases, including PubMed, Cumulative Index to Nursing and Allied Health Literature (CINAHL), Cochrane Library, EMBASE, and Web of Science from inception date until January 2024. The major terms used for searching included: (1) dysphagia; (2) dementia; (3) dysphagia treatment; and (4) swallowing function. An additional search was performed by tracking the citations of eligible papers. The search strategies for each database are detailed in Appendix A.

### Inclusion and exclusion criteria

#### Study designs

Case studies, quasi-experimental studies, observational studies, retrospective studies, open-label studies, and randomized controlled trials were included. Studies that did not involve original data, including meta-analyses, systematic reviews, and secondary studies were excluded. Studies in English or Chinese were eligible for inclusion.

#### Participants

Studies with participants clinically diagnosed with both dementia and dysphagia were included, regardless of the time of onset and severity of dementia and dysphagia. Studies with PLWD without dysphagia, and participants with concomitant dementia and other neurological diseases that were not analyzed separately from PLWD only were excluded.

#### Treatments

All dysphagia treatment methods, including non-invasive compensatory and rehabilitative strategies such as oromotor exercises, posture or diet modification, and enteral feeding strategies such as nasogastric (NG) tube and percutaneous endoscopic gastronomy (PEG) tube insertion, were included. Treatments that indirectly addressed swallowing function but had outcome measures assessing swallowing function were also included.

#### Outcome measures

Studies with any outcome measures that assessed swallowing function, including instrumental assessments, risk of aspiration or penetration, and pneumonia incidence, were included. Objective instrumental outcome measures were prioritized over subjective evaluations or self-reported outcomes in studies that reported several treatment outcomes.

### Data extraction and analysis

Data extraction was performed independently by the two authors. Data extracted from identified papers included: information of study (e.g., author, year of publishing), study design, demographic data of participants (e.g., age, patient characteristics, and severity of disease), treatment protocols (e.g., strategy, intensity, and duration), sample sizes and treatment outcomes. Qualitative analyses of the data were performed. A summary and patterns of the characteristics and findings of the included studies were synthesized and analyzed. The strengths and weaknesses of the evidence were critiqued, and the knowledge gaps were identified. Qualitative subgroup analysis was performed based on the type of treatment. If some data were not provided in the studies, an attempt was made to contact the authors, but if the data still could not be assessed, the study was excluded.

### Risk-of-bias assessment

The risk of bias in the included studies was assessed using the second version of the Cochrane risk-of-bias tool for randomized trials (RoB 2) [18] or the risk of bias in non-randomized studies (ROBINS) [19], depending on the study design. For randomized studies, domains assessed using RoB 2 included bias resulting from the randomization process, discrepancies between intended treatment and executed treatment, missing data, outcome measurement, and the selection of results reported [18]. For non-randomized studies, domains assessed using ROBINS included bias resulting from confounding, participant selection, treatment classification, discrepancies between intended treatment and executed treatment, missing data, outcome measurement, and the selection of results reported [19].

## Results

**Fig. 1:**
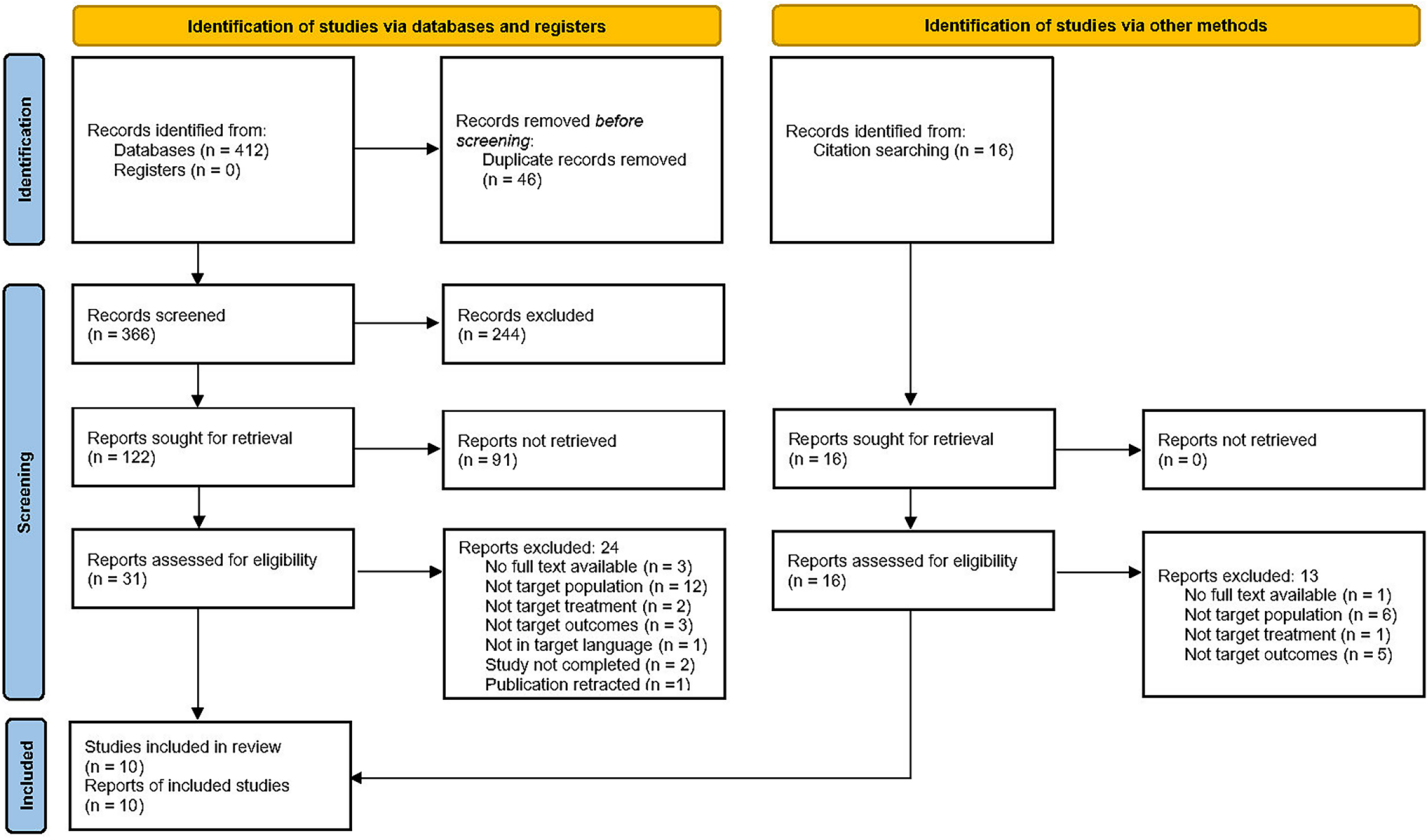
PRISMA flow chart of study identification, screening and selection

The process of the identification of studies is presented in Fig. 1. 428 studies were identified after the initial search from five electronic databases and other methods. Duplicates (*n* = 46) were identified and removed. The remaining studies (*n* = 382) were screened for eligibility. 335 studies were excluded during the title and abstract screening and 47 studies were screened in full text. 37 studies were excluded due to the following reasons: no full text available (*n* = 4); not targeted population (*n* = 18), in which some studies included persons without dementia or dysphagia, some included PLWD with other concomitant neurological diseases, some included PLWD with eating problems including dysphagia and refusal to eat, but did not provide independent analysis; not targeted outcomes (*n* = 8), in which some studies used outcome measurements not directly related to swallowing function, such as food intake and nutrition; not targeted treatment (*n* = 3), in which some studies focused on the provision of education and training on the medical staff, not direct treatment for PLWD; not in targeted language (*n* = 1), study not completed (*n* = 2), and publication retracted (*n* = 1). At last, 10 studies met all the inclusion criteria and were included in the analysis.

### Study characteristics

Five included studies were observational studies with prospective (*n* = 2) or retrospective (*n* = 3) designs, and five were RCTs. Four types of treatment were investigated (i.e., compensatory, rehabilitative, stimulation, and pharmacological treatments). The characteristics of the studies are summarized in Table 1.

**Table 1:** Characteristics of included studies

Study	Study type	Sample size [E/C]	Patient characteristics	Severity	Age^a^ (years)	Treatment (frequency) and comparison	Follow-up schedule	Swallowing-related outcome	Main findings
Cintra et al. (2014)	Observational prospective study	n = 67 [oral feeding n = 31/alternative feeding n = 36]	AD, stroke, dysphagia	Dementia: FAST 7A-7F Moderate to severe dysphagia	84.8 (7)	NGT vs oral feeding (6 months)	Every 3 months	Aspiration pneumonia incidence	Incidence of aspiration pneumonia: Alternative feeding > oral feeding (*p* = .006)**
Logemann et al. (2008)	RCT with crossover design	n = 711 (dementia only n = 282)	Dementia^2^ and/or PD, aspiration	Dementia: BANS-S range 7–28	50–95	Chin-down posture vs nectar-thickened liquid, honey-thickened liquid (single session)	Immediate	Aspiration	Aspiration in dementia-only group: Chin-tuck > nectar-thick liquids (*p* < .01) or honey-thick liquids (*p* < .0001)*** Nectar-thick liquids > honey-thick liquids (*p* < .0001)***
Robbins et al. (2008)	RCT with parallel design	n = 525 [chin-down n = 259/thickened-liquid n = 256] (dementia: chin-down n = 131/thickened liquids n = 129)	Dementia^b^ and/or PD, dysphagia	–	80.5	Chin-down posture vs thickened liquids (3 months)	3 months	Pneumonia incidence	No significant differences in 3-month cumulative incidence of pneumonia between groups
Masaki & Kawamoto (2019)	Retrospective cohort study	n = 253 [PEG n = 180/TPN n = 73] (dementia n = 102, PEG n = 57/TPN n = 45)	Dementia, dysphagia	Severe dementia	85.5	PEG vs TPN	404–823 days (median: 601 days)	Severe pneumonia incidence	Incidence of severe pneumonia: PEG > TPN (*p* = .01)* Better survival with PEG than TPN in persons living with severe dementia. No conclusions made regarding superiority of treatments
Bautmans et al. (2008)	RCT with crossover design	n = 15	AD, dysphagia	Severe dementia (MMSE median score: 8/30)	77–98	CSM vs. socializing visits (control) (three sessions)	One week (third session)	Maximal swallowing volume in a single movement	Swallowing capacity: significantly improved after one session of CSM (*p* = .01)* and after 1 week of treatment (*p* = .03)* compared to control
Chen et al. (2016)	Observational prospective cohort study	n = 30	Dementia, dysphagia	–	82.4(6.79)	Feeding intervention^c^ (pre- vs post-treatment) (3 months)	–	Kubota WST	Participants’ levels on the Kubota WST decreased (*p* < .001)***
Ye et al. (2024)	RCT with parallel design	n = 93 [48/45]	AD, dysphagia	Dysphagia: WST third degree of above, standard swallowing assessment score 18–46	80.72(7.1)	Stepwise swallowing training (5 × per week for 4 weeks) vs routine dysphagia care	Week 4	WST, standard swallowing assessment	Swallowing function: Treatment > control (WST *p* < .001)***, standard swallowing assessment *p* < .001)***
Nakashima et al. (2011)	RCT with parallel design; open-label	n = 60 [30/30] (dementia: 12/11)	Dysphagia, previous history of pneumonia, dementia	–	79.5[77–84.3]	Nicergoline vs Imidapril (6 months)	Once every month for 6 months	Simple 2-step swallowing provocation test, pneumonia recurrence	Improvement in swallowing reflex: Nicergoline > Imidapril (*p* < .05)* Increase in SP level: Nicergoline > Imidapril (*p* < .01)**
Yuen et al. (2022)	Retrospective cohort study	n = 764 [300/464] (dysphagia only: 111/274)	Dementia, dysphagia, behavioral feeding problem	Dementia: GDS stage 7, FAST level 7A-7F Mild to severe dysphagia	89(7.4)	CHF vs NG tube (1 year)	1 year	Pneumonia incidence	Pneumonia rates: CHF < NG tube feeding (*p* = .004)** NG tube feeding was not associated with pneumonia risk for participants with dysphagia only (*p* = 0.3)
Tang et al. (2017)	Retrospective study	n = 103 [53/50]	AD, dysphagia	Dementia: MMSE M = 14 Dysphagia: WST M = 4.15	73	SFT, simultaneous Mendelsohn maneuver and NMES and EMG-biofeedback therapy (1 h a day for 12 weeks) vs SFT only (45 circles per day for 12 weeks)	Week 4, Week 8, Week 12	WST, frequency and course of aspiration pneumonia	Improvement in WST: Treatment > Control (*p* < .05)* Significantly reduced frequency and shorter course of aspiration pneumonia in treatment group

Dash (–) represents data not reported

E/C, experimental/control, AD, Alzheimer’s disease; FAST, Functional Assessment Staging Tool; NGT, nasogastric tube; vs, versus; PD, Parkinson’s disease; BANS-S, Bedford Alzheimer Nursing Severity Scale; RCT, randomized controlled trial; PEG, percutaneous endoscopic gastrostomy; TPN, total parental nutrition; HR, hazard ratio; MMSE, Mini Mental State Examination; M, mean; CSM, cervical spine mobilization; WST, water swallow test; SP, serum substance P; GDS, Reisberg Global Deterioration Scale; CHF, careful hand feeding; SFT, swallowing function training, which was routine rehabilitation therapy that consisted of tongue exercises, pharynx and larynx exercises; NMES, neuromuscular electrical stimulation; EMG, electromyography

^a^Age are presented in mean (standard deviation), median [interquartile range] or range unless otherwise specified in Table 1

^b^Dementia participants in the study included Alzheimer’s, single or multistroke type or other nonresolving types

^c^Feeding intervention consisted of environmental modification, diet modification, posture modification, utensil modification and individualized feeding assistance

*p < 0.05, **p < 0.01, *** *p* < 0.001

### Participants characteristics

The included studies had a total of 2621 participants. Some studies included participants with Parkinson’s disease, stroke, or behavioral feeding problems. However, only data relevant to PLWD (*n* = 1360) were extracted for analysis in this review. Participants’ ages ranged from 50 to 98 years old, and the severity of dementia or dysphagia was heterogeneous across studies. Participants had different comorbidities (e.g., hypertension, diabetes mellitus).

### Treatments

#### Compensatory treatments: modification of feeding routes

Three included studies reported on compensatory treatments involving feeding route modifications, such as using NG tube [20, 21], PEG, or total parental feeding (TPN) [22]. Individuals with a functional gastrointestinal tract but deficits in other swallowing-related mechanisms may be recommended PEG or NG tube feeding for better nutrition management. In PEG feeding, a gastrostomy tube is inserted into the stomach through an endoscopy [23], while in NG tube feeding, a tube passes through the nose to the stomach to deliver food and liquids. Nutrients are injected intravenously into a person’s bloodstream in TPN, which is used when an individual’s gastrointestinal tract is not functioning [24].

Cintra et al. [20] studied 67 participants in a prospective study and concluded that oral feeding was better than alternative feeding as fewer participants in the oral feeding group had aspiration pneumonia. In the retrospective study of 253 participants, (102 PLWD), PEG and TPN were compared, and no conclusion was made regarding which enteral feeding method was superior [22]. Although PEG led to significantly longer survival time, it also led to a higher incidence of severe pneumonia. Yuen et al. [21] studied 764 PLWD with dysphagia and/or behavioral feeding problems in a retrospective study comparing NG tube and careful hand feeding (CHF) (385 PLWD with dysphagia only). NG tube feeding was associated with higher pneumonia rates compared to CHF in participants with coexisting dysphagia and behavioral feeding problem but not in participants with dysphagia alone [21]. In summary, no definitive conclusion could be made regarding the superiority of oral or alternative feeding routes. Although the above studies included a relatively large number of participants, most of the evidence came from retrospective studies, limiting control over potential confounders and making it difficult to draw definitive conclusions on causal relationships.

#### Compensatory treatments: other modifications

Five included studies investigated non-invasive modifications, such as postural, diet, environmental, and utensil modifications, and feeding assistance [21, 25–28]. Participants were treated with (1) diet modifications such as thickened liquids [27, 28] and softened food [26], (2) postural modifications such as chin-down posture [27, 28], cervical spine mobilization (CSM) [25], maintaining an upright seated position or a semi-reclining position for bedbound participants [26], (3) environmental modifications like regulating the temperature, humidity, ventilation, and minimizing distractions in the dining environment [26], (4) utensil modifications, including using colored forks and spoons with curved and larger handles for better distinguishment and physical manipulation of the utensils [26], and (5) feeding assistance such as CHF [21], reduced feeding speed, management of food placement in the oral cavity for better stimulation and keeping participants awake during mealtimes [26].

Logemann et al. [27] studied 711 participants (282 PLWD), in an RCT and concluded that using thickened liquids was better than chin-tuck posture, and honey-thickened liquids were better than nectar-thickened liquids in terms of reducing aspiration. However, a follow-up RCT [28] that investigated 525 participants (260 PLWD), demonstrated otherwise. Despite sharing participants with similar backgrounds as Logemann et al. [27], Robbins et al. [28] found that the 3-month cumulative pneumonia incidence was not significantly different between the chin-down posture group and the thickened-liquid groups, and between the nectar-thickened and honey-thickened-liquid groups. Hence, no definitive conclusions could be made on the superiority of chin-tuck posture or texture modification in improving swallowing safety based on these two studies.

Bautmans et al. [25] studied 15 participants in an RCT and found that CSM was effective in improving swallowing capacity. Chen et al.’s [26] prospective study of 30 participants showed that feeding intervention that included various compensatory strategies improved swallowing ability significantly. Yuen et al. [21] concluded that there was no significant difference in pneumonia risk between the CHF and NG tube feeding group. Taken together, CSM and feeding intervention appeared to be effective in improving swallowing ability. While most of the included studies investigated compensatory treatment methods, most of the treatments were only addressed by one study, making it hard to draw solid conclusions regarding treatment effectiveness.

#### Rehabilitative treatments

One included study examined the effects of rehabilitative treatment focusing on exercising swallowing muscles. Ye et al. [29] studied 93 participants in an RCT evaluating the effects of a stepwise swallowing training (SST), which consisted of video-based exercises targeting progressive movements for different swallowing muscle groups (i.e., lip, cheek, tongue, mandible, and neck) and found that SST was effective in improving swallowing function.

#### Stimulation treatments

One included study investigated the effect of simultaneous Mendelsohn maneuver and neuromuscular electrical stimulation (NMES) with electromyographic (EMG)-biofeedback therapy in addition to rehabilitation training exercises on the swallowing function of participants with AD [30]. Skin electrodes were attached to participants and electrical stimulation was provided via VitalStim at a frequency of 80 Hz, wave width of 700 μs, and amplitude of 0 to 25 mA [30]. The stimulation intensity was increased until participants reported a “grabbing” sensation [30] (p.2). Participants were able to visualize their pharyngeal muscle activity during the Mendelsohn maneuver via EMG [30]. Tang et al.’s [30] retrospective study of 103 participants concluded that swallowing training combined with NMES and EMG-biofeedback was more effective in reducing frequency and shortening the course of pneumonia than swallowing training alone.

#### Pharmacological treatments

One included study, Nakashima et al. [31], an open-label RCT that studied 60 participants (23 PLWD), investigated whether nicergoline could improve dysphagia. Nicergoline is an ergot derivative widely prescribed to persons with balance or cerebrovascular disorders and can induce many effects, including increasing dopaminergic neurotransmission [32]. Substance P is a neurotransmitter that enhances the swallowing reflex, and the reduction in substance P secretion has been associated with reduced swallowing reflex [31]. Since nicergoline is known to promote the function of dopaminergic neurotransmitters, which can cause substance P secretion, it was hypothesized by Nakashima et al. [31] to be able to improve the swallowing reflex. In the study, nicergoline was compared with imidapril, an angiotensin-converting enzyme inhibitor that has been known to improve swallowing reflex by raising substance P levels in persons with dysphagia [33] and lowering the risk of pneumonia [34]. This study showed that nicergoline was more effective than imidapril in increasing substance P and improving swallowing reflex.

### Treatment duration and follow-up schedule

Treatment duration ranged from one session to 823 days. Except for several retrospective studies, most of the studies investigated the short-term (3–6 months) treatment effects due to unstable medical conditions of frail older participants.

### Outcome measures

Five studies measured pneumonia incidence or recurrence, through caregivers’ reports, chest radiography, or physicians’ clinical diagnosis [20–22, 28, 31]. One study measured the frequency and course of aspiration pneumonia [30]. Five studies measured participants’ swallowing function using the five-level Water Swallowing Test [26, 29, 30], Standard Swallowing Assessment [29], and assessing the maximal swallowing capacity in a single movement [25]. One study measured aspiration occurrence using the videofluoroscopic swallow study (VFSS) [27] and one study measured swallowing reflex in a 2-step swallowing provocation test using a nasal catheter [31].

### Risk-of-bias assessment

**Fig. 2:**
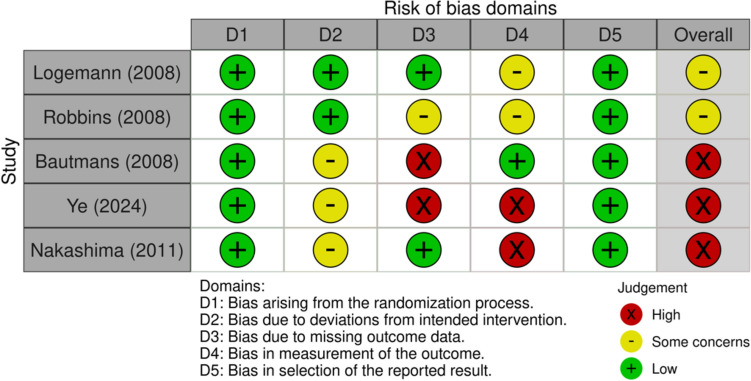
Risk-of-bias graph for randomized studies

**Fig. 3:**
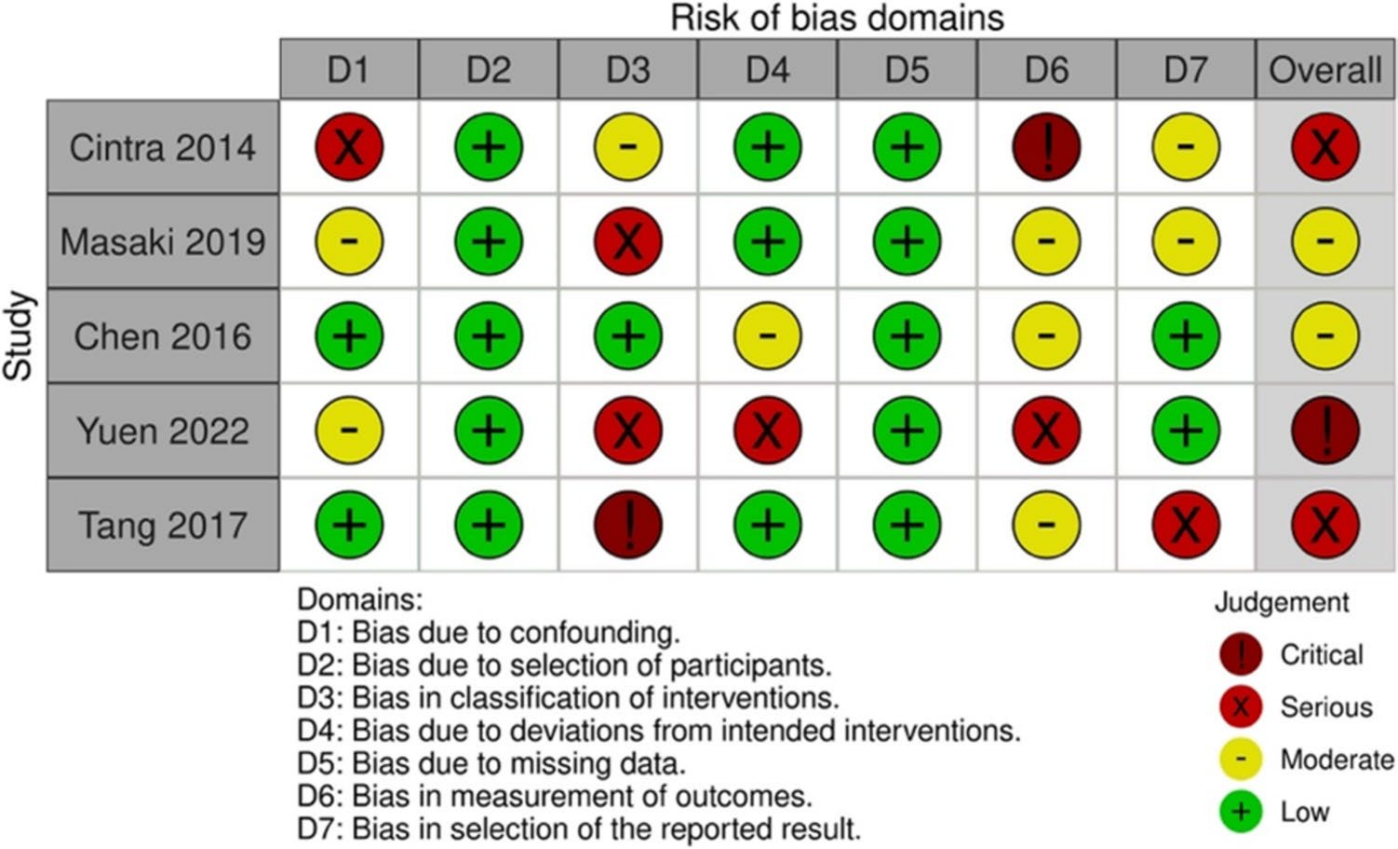
Risk-of-bias graph for non-randomized studies

The results of the risk-of-bias assessment are presented in Figs. 2 and 3. All the studies had at least some concerns regarding the overall risk of bias. For RCTs, the most common concerns were (1) bias due to insufficient blinding in treatment delivery, and (2) bias in outcome measurement as assessors were not blinded to treatment allocation. For non-RCTs, the most common concerns included (1) bias in treatment classification as decisions were likely affected by knowledge of risks and outcome, (2) bias due to deviation from intended interventions due to insufficient blinding to treatment and difficulty in monitoring the adherence and quality of caregiver-delivered treatments. The non-RCT with a particularly high risk of bias included Cintra et al. [20] and Yuen et al. [21]. The former study was at high risk of bias in the measurement of outcomes as the outcome assessors of the study, the primary caregiver, were not blinded to treatment. This may increase the risk of bias due to placebo effect. There was also a high risk of bias due to confounding, as the Kaplan–Meier analysis did not take the crossover between groups (e.g., changing of tubes, changing of feeding mode) into consideration, thus favoring the oral feeding group in terms of survival [20]. The study by Yuen et al. [21] had a high risk of bias due to deviation from the intended intervention. Although CHF plans were administered and periodically reviewed by a multidisciplinary team of medical staff, participants’ primary caregivers were allowed to provide feeding assistance if they wished to, causing possible nonadherence to the intended treatment. Moreover, all analyses were conducted based on the original feeding mode, regardless of any changes during treatment or follow-up period. There was also a high risk of bias in outcome measurement, as the outcome measurement was done by the treating physician’s diagnosis of new pneumonia occurrence, but whether the occurrence was directly related to aspiration could not be verified.

## Discussion

This systematic review explored the effects of dysphagia treatments in persons living with any type of dementia. Overall, our results suggested that dysphagia treatments may be effective in ameliorating dysphagia in PLWD. However, cautions are needed when interpreting the results due to (1) high heterogeneity in study designs and treatment protocols, (2) inconsistent outcome measurements across studies, (3) co-administration of different treatments within a study cohort, and (4) limited number of studies available for each treatment. Therefore, definitive conclusions could not be made about the type of dysphagia treatment most beneficial for PLWD. The risk-of-bias assessment results showed that most of the studies were subjected to a high risk of bias, indicating that quality studies investigating dysphagia treatment efficacy in PLWD are still highly inadequate.

Among the 10 included studies, more studies investigated compensatory methods compared to rehabilitative methods. Such phenomenon may be related to dementia characteristics, such as difficulty in understanding and following more complex instructions [27] demanded from rehabilitative methods. Logemann et al. [27] reported that treatment adherence was lower among PLWD compared to participants with Parkinson’s disease for chin-down posture, which required a higher cognitive and comprehension ability to follow, whereas PLWD’s adherence to honey-thickened-liquid treatment was higher. Such findings may be suggestive of passive treatments being better than active treatments for PLWD. Nonetheless, some studies suggested otherwise, hence the controversy about the suitability of active or passive treatments remains. Bautmans et al. [25] suggested that CSM involved the painless passive movement of the head, without needing PLWD’s active participation, substantiating that such procedures are thus feasible and suitable for frail PLWD with severe cognitive decline. Contrastively, while Ye et al. [29] acknowledged that rehabilitative treatments may be challenging for persons living with AD due to cognitive decline, they highlighted the importance of active participation in treatment, as participants showed significant improvements in swallowing function through learning and performing swallowing muscle exercises, refuting the traditional belief that learning capacity is limited with age [35]. Difficulty in concluding the superiority of the type of treatment may also be influenced by the heterogeneity of study designs. Some studies proposed a mix of passive treatments with rehabilitative exercises. In Tang et al.’s [30] study, passive stimulation treatment using NMES and EMG-biofeedback with swallowing rehabilitation training was shown to be more effective than performing the latter alone in improving swallowing function. Overall, no conclusions could be drawn on the superiority of compensatory or rehabilitative swallowing treatments.

Different types of dementia may present different swallowing difficulties. AD particularly affects sensory aspects, resulting in a prolonged oral phase. Vascular dementia affects motor aspects, causing mastication and bolus manipulation difficulties [36]. Frontotemporal dementia may lead to feeding problems such as overeating and rapid eating [37]. Finally, Lewy body dementia particularly disturbs oesophageal mobility [5]. Given the dementia-induced sensory, motor, and cognitive changes that affect swallowing in different ways and to different extents, varying approaches to treatment may be required. Moreover, when considering treatment for PLWD, many variables (e.g., comorbidities, cognitive and physical abilities, caregivers’ age and abilities) other than dysphagia itself, need to be considered. This may be a plausible reason for the lack of dysphagia treatment specific for PLWD to date. Some studies promoted treatment personalization, such as mixing treatment methods to suit each PLWD’s needs. In Chen et al.’s [26] study, instead of having one treatment for all participants, treatment methods were selected according to participants’ personalities, interests, and eating preferences, which brought them increased confidence and quality of life (QoL) in eating. Logemann et al. [27] provided evidence for a combination of treatment methods as they demonstrated the elimination of aspiration of thin liquid with one or more of the three treatments in almost half of the participants. Tang et al. [30] also found that combined NMES and swallowing rehabilitation training was more beneficial than rehabilitation training alone. Moreover, given the nature of dementia being a progressive disease, the display of PLWD’s clinical profiles can change continuously. Some studies reported that a change in treatment regimen was necessary due to a change in the PLWD’s medical condition [20, 21]. Therefore, regular reviews are essential to monitor the effectiveness of dysphagia treatment for PLWD. Individualized and comprehensive dysphagia treatments for PLWD should especially be considered, as the clinical profiles of PLWD are diverse and may change throughout the progression of the disease. Their needs and challenges may therefore change overtime. Individualized treatment that takes into consideration of individual needs and differences can reduce their anxiety towards the changes and promote the well-being of PLWD.

A shift in focus of swallowing treatment provision was observed in the studies published in more recent years [21, 26, 29]. There was a common and growing emphasis on interprofessional collaboration, where all-rounded treatments were delivered by multidisciplinary professionals (e.g., nurses, nutritionists, therapists, and social workers) to perform a comprehensive care plan tackling the physical, cognitive, and socio-emotional difficulties accompanying dementia. Insights from multidisciplinary teams could help with tailor-making treatment plans that best suit the PLWD’s individual needs for optimal treatment outcomes [38]. Ye et al. [29] investigated SST delivered by nurses. The significant result demonstrated the feasibility and effectiveness of other trained professionals delivering swallowing-related treatment, offering a potential solution to the global shortage of speech therapists. Multidisciplinary team management may also reduce the number of deaths and functional decline among older persons [38]. Apart from healthcare professionals, several studies highlighted the importance of caregiver involvement in PLWD’s dysphagia management. Caregivers helped with meal monitoring [27], providing feeding assistance during CHF [21], and even delivering treatment upon hospital discharge [29]. These findings suggested the potential for caregiver-delivered treatment for PLWD. Moreover, besides solely focusing on swallowing safety, the importance of PLWD’s QoL was also recognized in many studies, especially regarding their diet and feeding-mode preference. Participants reported disliking thickened liquids [27], and swallowing function was improved when emphasis was put on participants’ autonomous eating in a feeding intervention [26]. Taken together, dysphagia management in PLWD appeared to be most effective when a multidisciplinary team of healthcare professionals, caregivers, and PLWD are involved, as decisions made can be understood and reviewed by different stakeholders to ensure effective management and sufficient support to PLWD and their caregivers [39].

A take-home message from the included studies is that public education on swallowing-related knowledge is still lacking. For instance, regarding the relationship between liquid viscosity and swallowing safety, the common belief was “the thicker the liquid, the safer the swallow” so the thickening of liquids was often promoted as an oropharyngeal dysphagia treatment [27, 40]. However, this belief was refuted by Robbins et al. [28] as the incidence of pneumonia was lower in the nectar-thick liquid group compared to the honey-thick liquid group. Second, in some countries like Japan, TPN was sometimes misguidedly used in older persons with dysphagia who have a functional gastrointestinal tract [22]. It is important to educate that TPN is not an alternative to PEG feeding, and PLWD’s clinical conditions, preferences, and QoL must be considered when making feeding-mode decisions. Moreover, caregiver education is equally important. As caregivers often help with monitoring food intake and executing prescribed treatment, especially dietary modifications, their concordance and compliance to treatment are vital for the treatment process and the possible improvement in PLWD’s dysphagia. It has been reported that caregivers played a key role in informing staff about PLWD’s signs of swallowing difficulties, which eventually led to a change in treatment prescription [41]. Hence, caregivers’ knowledge about dementia, dysphagia symptoms, and diet modifications can increase their support towards PLWD’s meal experience. Previous research has also demonstrated that providing decision aids about feeding options for caregivers of persons living with AD could increase their knowledge and aid them in feeding-mode decision-making [42]. They should also be taught to pay attention to the possible adverse events during swallowing (e.g., fatigue, coughing during swallowing) to ensure PLWD’s swallowing safety upon discharge from hospitals or when conducting dysphagia treatments at home. It was also reported that some caregivers felt distressed due to the conflict between having to adhere to the medical staff’s treatment prescription and managing PLWD’s negative reactions (e.g., refusal to eat, discomfort) towards modified diets, which are commonly associated with being less appetizing [41]. PLWD’s negative reactions may lead to caregivers to frustration in executing the prescribed treatment and affect their adherence to treatment delivery. Hence, education on strategies to interact with PLWD and to cope with the impacts of negative emotional reactions from PLWD on the caregiver’s mental health, and the importance of adherence to treatment regimens is important for quality caregiver-delivered treatment.

This review has several limitations. First, no included prospective studies investigated the long-term (i.e., above 2 years) treatment effectiveness. As dementia is degenerative, more studies are needed to evaluate the long-term interaction between dysphagia treatment and the changing clinical condition of PLWD. Furthermore, the small number of RCTs included limited the ability to conclude treatment efficacy, and findings may not be representative of the entire PLWD population. The high heterogeneity in study designs, sample populations, and outcome measurement methods also caused difficulty in drawing conclusions with confidence. Future studies should specify the inclusion and exclusion criteria for the study population clearly to enhance external reliability and facilitate the comparison of treatment effects across studies. Importantly, our findings call for a consensus on the optimal outcome measurements for PLWD to facilitate evaluation of treatment efficacy across studies. Given the complexity and degenerative nature of dementia, it is essential to consider the domains in the International Classification of Functioning Disability and Health (ICF) [43] when deciding the optimal outcome measurements for PLWD. Instrumental assessments of swallowing, such as VFSS or fiberoptic endoscopic evaluation of swallowing (FEES), should be prioritized, as they can reflect the deficits during swallowing with relatively low measurement errors than clinical bedside assessment of swallowing [44]. In addition to instrumental assessment on swallowing, subjective reports from the caregivers may provide valuable insights on the impacts of dysphagia on the PLWD’s daily life activities and participation. It should be noted that while self-report questionnaires may be useful in understanding the impact of dysphagia on quality of life from the patient’s perspective, they should be interpreted with cautions as they may not be reliable depending on the severity of cognitive impairment. Finally, our results from the risk of bias assessment showed that most of the included studies had some risk of bias. We found that some non-RCTs had high risks of bias related to blinding of assessment or treatment delivery and deviation from intended treatment and we have detailed the reasons for these biases. It is important that these risks of bias should be taken into consideration when interpreting the findings from this review.

Taken together, our review provided a summary on the clinical efficacy of dysphagia treatments for PLWD, which indicated that there is a lack of conclusive evidence and highlighted several directions for further studies. The included studies explored dysphagia treatments in different settings (e.g., hospitals, nursing homes) but the benefits of dysphagia treatment conducted at home by caregivers remain unclear. Further research in this direction can provide evidence on the efficacy and feasibility of caregiver-delivered treatment, as caregivers have the closest contact with PLWD and may achieve greater treatment efficacy through more frequent treatment, especially for rehabilitative treatments. Moreover, more studies are needed to investigate the long-term or maintenance effects of treatment due to the progressive nature of dementia. Finally, regarding the study methodology, future studies should specify the eligibility criteria of study sample, include instrumental assessment of swallowing and potentially reports from caregivers on the impact of dysphagia on the patient’s activity and participation in life, and employ methods to minimize bias to facilitate the evaluation on the efficacy of different dysphagia treatments for PLWD.

## Conclusions

In conclusion, this systematic review showed that different types of dysphagia treatments can potentially improve PLWD’s swallowing function, but efficacy is still dependent on individual clinical profiles. The level of current evidence is low, with a lack of RCTs and high heterogeneity in study methodology. In the future, more RCTs with homogenous sample populations and long-term follow-ups are needed to draw more definitive conclusions on dysphagia treatment efficacy for PLWD.

## Supplementary Information

Below is the link to the electronic supplementary material.

Supplementary file1 (DCOX 738 KB)

## Data Availability

The data that support the findings of this study are available from the corresponding author, IC, upon reasonable request.
