# Supplementary material for: A systematic review on dysphagia treatments for persons living with dementia
Source: Eur Geriatr Med. 2024 Nov 29;15(6):1573–85. doi: 10.1007/s41999-024-01107-6 (PMC11632040; doi:10.1007/s41999-024-01107-6)
Supplement: Supplementary file 1 — Supplementary file1 (DCOX 738 KB) [file 41999_2024_1107_MOESM1_ESM.docx]

**Appendix A: Search strategies**

1. **PubMed**

(dysphagia OR “swallowing disorders” OR deglutition OR aspiration) AND (dementia) AND (dementia OR alzheimer’s OR vascular OR “lewy body” OR frontotemporal OR “cognitive impairment”) AND (dysphagia treatment OR intervention OR therapy) AND (swallowing function OR swallowing safety OR swallowing efficiency OR quality of life)

Filters: case reports, clinical study, clinical trial, comparative study, controlled clinical trial, observational study, randomized controlled trial

1. **CINAHL**

(dysphagia OR “swallowing disorders” OR deglutition OR aspiration) AND (dementia OR alzheimer’s OR vascular OR “lewy body” OR frontotemporal OR “cognitive impairment”) AND (dysphagia treatment OR intervention OR therapy) AND (swallowing function OR swallowing safety OR swallowing efficiency OR quality of life)

1. **Embase**

(dysphagia OR “swallowing disorders” OR deglutition OR aspiration) AND dementia AND (dementia OR alzheimer’s OR vascular OR “lewy body” OR frontotemporal OR “cognitive impairment”) AND (dysphagia treatment or intervention or therapy) AND (swallowing function OR swallowing safety OR swallowing efficiency OR “quality of life”)

Filters: Full Text, Human, English Language

1. **Cochrane Library**

(dysphagia OR “swallowing disorders” OR deglutition OR aspiration) AND (dementia OR alzheimer’s OR vascular OR “lewy body” OR frontotemporal OR “cognitive impairment”) AND (dysphagia treatment OR intervention OR therapy) AND (swallowing function OR swallowing safety OR swallowing efficiency OR quality of life)

(Word variations have been searched)

Filter: Trials

1. **Web of Science**

(dysphagia OR “swallowing disorders” OR deglutition OR aspiration) AND (dementia) AND (dementia OR alzheimer’s OR vascular OR “lewy body” OR frontotemporal OR “cognitive impairment”) AND (dysphagia treatment OR intervention OR therapy) AND (swallowing function OR swallowing safety OR swallowing efficiency OR quality of life)
